# Evolutionary analysis of the highly dynamic *CHEK2 *duplicon in anthropoids

**DOI:** 10.1186/1471-2148-8-269

**Published:** 2008-10-02

**Authors:** Claudia Münch, Stefan Kirsch, António MG Fernandes, Werner Schempp

**Affiliations:** 1 Institute of Human Genetics and Anthropology, University of Freiburg, Breisacher Str. 33, 79106 Freiburg, Germany

## Abstract

**Background:**

Segmental duplications (SDs) are euchromatic portions of genomic DNA (≥ 1 kb) that occur at more than one site within the genome, and typically share a high level of sequence identity (>90%). Approximately 5% of the human genome is composed of such duplicated sequences. Here we report the detailed investigation of *CHEK2 *duplications. *CHEK2 *is a multiorgan cancer susceptibility gene encoding a cell cycle checkpoint kinase acting in the DNA-damage response signalling pathway. The continuous presence of the *CHEK2 *gene in all eukaryotes and its important role in maintaining genome stability prompted us to investigate the duplicative evolution and phylogeny of *CHEK2 *and its paralogs during anthropoid evolution.

**Results:**

To study *CHEK2 *duplicon evolution in anthropoids we applied a combination of comparative FISH and *in silico *analyses. Our comparative FISH results with a *CHEK2 *fosmid probe revealed the single-copy status of *CHEK2 *in New World monkeys, Old World monkeys and gibbons. Whereas a single *CHEK2 *duplication was detected in orangutan, a multi-site signal pattern indicated a burst of duplication in African great apes and human. Phylogenetic analysis of paralogous and ancestral *CHEK2 *sequences in human, chimpanzee and rhesus macaque confirmed this burst of duplication, which occurred after the radiation of orangutan and African great apes. In addition, we used inter-species quantitative PCR to determine *CHEK2 *copy numbers. An amplification of *CHEK2 *was detected in African great apes and the highest *CHEK2 *copy number of all analysed species was observed in the human genome. Furthermore, we detected variation in *CHEK2 *copy numbers within the analysed set of human samples.

**Conclusion:**

Our detailed analysis revealed the highly dynamic nature of *CHEK2 *duplication during anthropoid evolution. We determined a burst of *CHEK2 *duplication after the radiation of orangutan and African great apes and identified the highest *CHEK2 *copy number in human. In conclusion, our analysis of *CHEK2 *duplicon evolution revealed that SDs contribute to inter-species variation. Furthermore, our qPCR analysis led us to presume *CHEK2 *copy number variation in human, and molecular diagnostics of the cancer susceptibility gene *CHEK2 *inside the duplicated region might be hampered by the individual-specific set of duplicons.

## Background

Segmental duplications (SDs) are euchromatic portions of genomic DNA (≥ 1 kb) that occur at more than one site within the genome and typically share a high level of sequence identity (>90%) [1]. Both *in situ *hybridization and *in silico *analyses have shown that ~5% of the human genome is composed of duplicated sequences [2-4]. Duplications that can be traced to an ancestral or donor location are named duplicons. Based on a neutral model of genome evolution [5], duplicons with approximately 10% sequence divergence correspond to duplication events that have occurred 30–40 million years ago (MYA), i.e. before the radiation of Old World monkey and hominoid species [6]. Furthermore, a conspicuous bias of interchromosomal SDs toward pericentromeric regions, or euchromatin/heterochromatin transition regions in general, was detected [7,8]. Fine-scale analyses of pericentromeric regions disclosed a two-step model for the formation of such dynamic regions. An initial pericentromeric "seeding" event followed by subsequent exchange ("swapping") of duplicon blocks between pericentromeric regions has been proposed [9,10]. Both homologous and non-homologous processes were shown to be involved in "seeding" and "swapping" of pericentromeric SDs in human and great ape genomes [9-13] (for review see [14]). The duplicative architecture of human and higher primate genomes has been shown to be a major force promoting rapid evolutionary turnover [15]. Although the predisposition to expansion of interspersed segmental duplication is common to human and great apes, it appears that many species-specific duplication events have taken place at different regions of their genomes. Interestingly, two independent approaches determined the fraction of species-specific SDs in chimpanzee and human to be ~30%, while ~66% of SDs seem to be shared between both species [16,17]. Thus, species-specific SDs are thought to have contributed to a larger extent to the genetic difference between chimpanzee and human than single-base mutations [17]. Moreover, SDs seem to be sites of recurrent large-scale structural variations [18-20] and it has been estimated that ~20% of SDs are polymorphic within the human and chimpanzee genome [17].

Interestingly, among all human chromosomes the Y chromosome has the highest relative SD content [2,6,16,21-23]. Recently, we have analyzed 866 kb of Y-chromosomal non-palindromic SDs delineating the four euchromatin/heterochromatin transition regions in Yp11.2/Yp11.1, Yq11.1/Yq11.21, Yq11.23/Yq12 and Yq12/YPAR2 [24,25].

Here we report the detailed investigation of the *CHEK2 *duplicons, one of which is embedded within the analyzed Yq11.1/Yq11.21 SD cluster. The ancestral duplicated region, containing the proximal part of the functional *CHEK2 *gene (CHK2 checkpoint homolog S.pombe) and the distal part of *TTC28 *(tetratricopeptide repeat domain 28), is located in 22q12.1 [10,25-27]. *CHEK2 *has been shown to be a multiorgan cancer susceptibility gene [28]. Interestingly, CHK2, the protein encoded by *CHEK2*, is a cell cycle checkpoint kinase acting in the DNA-damage response signalling pathway [27]. Cell cycle checkpoints monitor the structural integrity of chromosomes before their progression through crucial cell cycle stages. CHK2 homologues were found in yeast and higher eukaryotes [29-33] indicating an important role throughout eukaryotic evolution in controlling the integrity of the genome. The continuous presence of the *CHEK2 *gene in all eukaryotes [34] and its important role in maintaining genome stability [27] prompted us to investigate the duplicative evolution and phylogeny of *CHEK2 *and its paralogs during anthropoid evolution. We applied a combination of comparative FISH and *in silico *analyses. In addition, we used inter-species quantitative PCR for further validation and for detection of intra-species specific *CHEK2 *copy number variations.

## Methods

### Y-chromosomal cosmid library screening

We screened the LLOYNC03 "M" (Lawrence Livermore National Laboratory) Y-chromosomal library (5.5xY chromosome coverage) for *CHEK2 *duplicon containing cosmids using a Y-chromosomal probe generated by the following PCR-conditions: 95°C for 5 min, 40 cycles of 94°C for 30 sec, 62°C for 30 sec and 72°C for 1 min and finally 72°C for 5 min. The following primers were used to generate a 575 bp probe: IP-cos-SD1-for 5'-ACCCCCTTAGTAGCGTCCTTAGCTC-3' and IP-cos-SD1-rev 5'-ACCACCGGAGTTTCACAAAGAAAGT-3'. The gel-purified probe was radioactively labelled according to the 'random priming' protocol [35]. Prehybridization and hybridization of the high density gridded filters were carried out for 18 hrs at 42°C according to the manufacturer's protocol. Final filter-washing was carried out in 1%SDS/2xSSC solution for 1 hr at 65°C. Primers located proximal and distal of the derivative Y-chromosomal *CHEK2 *duplicon were used for PCR (conditions see above) to identify cosmids containing the entire duplicon:

PB-cos-SD1-for 5'-AGCGCAAATTGCAGAATTACAAAGA-3', PB-cos-SD1-rev 5'-GGTTAGAGAGGATAAGCCGCATGTT-3', DB-cos-SD1-for 5'-GATCCCGCACATTTGTTCATTAGAG-3', DB-cos-SD1-rev 5'-CAAAAGCTTGAATTCTGTGCCTCAGT-3'

### Detection of derived CAGGG repeat sequences in the Y-chromosomal cosmid LLOYNC03 "M" 22E01

We used RepeatMasker http://www.repeatmasker.org/ and Tandem Repeats Finder [36] to search for CAGGG repeat sequences within the Y-chromosomal *CHEK2 *duplicon containing cosmid LLOYNC03"M" 22E01. Both analyses failed to identify CAGGG repeat sequences in the derivative *CHEK2 *duplicon and in both adjacent derivative duplicons, which are the *IGL@*- (Immunoglobulin lambda@ locus-) and the *NHEDC1*- (Na^+^/H^+ ^exchanger domain containing 1-) duplicons. Nevertheless, RepeatMasker and Tandem Repeats Finder analysis revealed the presence of extensive CAGGG repeat sequences within the ancestral *IGL@ *locus in 22q11.21 (NT_011520: 1994740–2060046). Subsequent pairwise alignment of both ancestral and the derivative Y-chromosomal *IGL@ *loci disclosed a more diverged CAGGG repeat like sequence. By using the DnaSP Ver.4.10.9 software 72% nucleotide sequence identity was detected between the CAGGG repeats present within the ancestral *IGL*@-duplicon (NT_011520: 2033670–2035327) and the derivative CAGGG repeats in Yq11.1/Yq11.21 (NT_113819: 398233–403886).

### Blood samples and cell lines

The following blood samples from non-related individuals were used for qPCR analysis: blood samples from the chimpanzees (*Pan troglodytes*, PTR) Max (LN: 444) and Fritz (LN: 776, wild-born) were obtained from the Zoologisch-Botanischer Garten Wilhelma Stuttgart (Germany). The blood sample from chimpanzee Marcel (LN: 39) was obtained from TNO Primate Centre Rijswijk (Netherlands). Lowland gorilla (*Gorilla gorilla gorilla*, GGO) blood samples of Jangu (LN: 315), Gaidi (LN: 916, wild-born) and Fritz (LN: 673, wild-born) were obtained from the Zoo Wuppertal, the Zoo Leipzig and the Tiergarten Nürnberg, respectively. Two blood samples of the Bornean orangutans (*Pongo pygmaeus pygmaeus*, PPY) Thai (LN: 866) and Napoleon (LN: 1005) were obtained from the Zoo Duisburg (Germany) and the Zoo Studen (Switzerland), respectively. Blood samples from two rhesus macaque individuals (*Macaca mulatta*, MMU; LN: 1053 and 1054) and one hamadryas baboon individual (*Papio hamadryas*, PHA; LN: 496) were obtained from the Deutsches Primaten Zentrum Göttingen (Germany).

Skin tissue of a pig-tailed macaque (*Macaca nemestrina*, MNE) was provided by the Deutsches Primaten Zentrum Göttingen (Germany) and was used to establish a fibroblast cell line. For each species one of the above listed blood samples and the fibroblast cell line were used for FISH analysis. Lymphoblastoid cell lines of the white-cheeked crested gibbon (*Nomascus leucogenys*, NLE) and the common marmoset (*Callithrix jacchus*, CJA) were kindly provided by S. Müller (Munich) and were used for FISH analysis.

### Fluorescence in situ hybridization (FISH)

FISH analysis of metaphase spreads derived from lymphocytes or lymphoblastoid and fibroblast cell lines from non-related human (*Homo sapiens*, HSA) and non-human primate males was performed. Prior to FISH, the slides were treated with RNase followed by pepsin digestion as described [37]. FISH was carried out following the protocol described previously [38]. Chromosome *in situ *suppression was applied to clones from the human fosmid library WI-2 (WI2-1621D20, WI2-819H21) and from the Y chromosome specific cosmid library LLOYNCO3"M" (LLOYNCO3"M"22E01). Human whole-chromosome painting (WCP) libraries [39] were used to unequivocally assign hybridizing signals to orthologous regions in lesser apes, Old World monkeys and New World monkeys. pMR100, a mouse-derived rDNA-containing plasmid, was used to tag the Old World monkey marker chromosome. After FISH the slides were counterstained with DAPI (4',6-diamidino-2-phenylindole; 0.14 μg/ml) and mounted in Vectashield (Vector Laboratories). Preparations were evaluated using a Zeiss Axiophot epifluorescence microscope equipped with single-bandpass filters for excitation of red, green, and blue (Chroma Technologies, Brattleboro, VT). During exposures, only excitation filters were changed allowing for pixel-shift-free image recording. Images of high magnification and resolution were obtained using a black-and-white CCD camera (Photometrics Kodak KAF 1400; Kodak, Tucson, AZ) connected to the Axiophot. Camera control and digital image acquisition involved the use of an Apple Macintosh Quadra 950 computer.

### Phylogenetic analysis

FASTA formatted sequence files used to generate phylogenetic trees were extracted from the corresponding GenBank accession numbers. Sequence alignments were built by using CLUSTALW (version 1.82) [40], and neighbor-joining phylograms created by using MEGA (Molecular Evolutionary Genetic Analysis) v4.0 http://www.megasoftware.net[41]. Neighbor-joining analysis was used with complete deletion parameters and bootstrap (1,000 iterations) to provide confidence of each branching point in the phylogenetic trees. Neighbor-joining methods were chosen as they are amenable to calculating divergence times between sequence taxa. We estimated the number of substitutions per site per year by correcting the divergence times for multiple substitutions using Kimura's two-parameter model [42]. As the rates of nucleotide substitution vary for pseudogenic sequences, the rate of nucleotide substitution was calibrated based on orthologous sequence comparisons using a divergence of 25 Mya for macaque-human divergence [43]. Duplication timing events were calculated by applying the equation r = k/2 T [44], where r is the rate of nucleotide changes per bp per yr, k is the distance calculated between the ancestral and paralogous sequences, and T is the time of divergence of the molecules.

### Quantitative PCR

Interspecies quantitative PCR was carried out using primers specific for *CHEK2 *exon 14. Primers were designed with the assistance of the Promega Plexor Primer Design Software. The following primer sequences were used: *CHEK2*-exon14-F 5'-GGACCTTGTCAAGAAGTTGTTGGT-3', *CHEK2*-exon14-R 5'-GGTGTCTTAAGGCTTCTTCTGTCGTA-3', *CFTR*-F 5'-CGCGATTTATCTAGGCATAGGC-3' and *CFTR*-R 5'-TGTGATGAAGGCCAAAAATGG-3'

We used the ABI Prism 7900 HT system (Applied Biosystems) for real time detection. Reactions contained 0.25 μM of each primer and 5 μl of QuantiTec SYBR^® ^Green PCR Master Mix (Quiagen) in a total of 10 μl. Assays included DNA standards at a final concentration of 5.0, 2.5, 1.25, 0.625 and 0.3125 ng/μl, a no-template control, or 1 ng/μl of the species DNA in two replicates. Cycling conditions were 50°C for two minutes, 95°C for 15 minutes, and 40 cycles of 95°C for 15 sec, 58°C for 30 sec and 72°C for 30 sec.

To avoid the generation of non-specific products, a melting curve analysis of products was routinely undertaken following the amplification. A standard curve was constructed by plotting the cycle number (Ct), at which the amount of target in standard dilutions reaches a fixed threshold, against the log of the amount of starting target. For standard curve construction genomic DNA from the rhesus macaque MMU#13577 was used as a *CHEK2 *single copy reference. The *CHEK2 *single copy status in the rhesus macaque genome was verified by both FISH and *in silico *analysis. Absolute quantification of copy number in the different species was subsequently done by interpolation of the threshold cycle number (Ct) against the corresponding standard curve. Copy numbers of the test genes in primate samples were normalised to the copy number of the *CFTR *gene (cystic fibrosis transmembrane conductance regulator), which serves as a control representative of a single gene per haploid genome [45]. *CFTR *primers perfectly match the *CFTR *gene in all targeted species genomes. The ratio of the *CHEK2 *copy number to *CFTR *copy number in each sample normalised the results with respect to differing starting quantity and quality of the template DNA in each reaction [46]. Standard errors of the normalised *CHEK2 *copy numbers were calculated from the standard deviations of the values of the *CFTR *and *CHEK2 *genes using the formula provided by the user menu (ABI Prism 7700 Sequence Detection System, User Bulletin no.2 1997, p.34). Comparisons between the mean values were performed using the Student unpaired *t*-test. A *P*-value <0.001 was considered significant.

## Results and discussion

### Identification and comparative FISH of a *CHEK2 *duplicon containing Y-chromosomal cosmid probe

**Figure 1:**
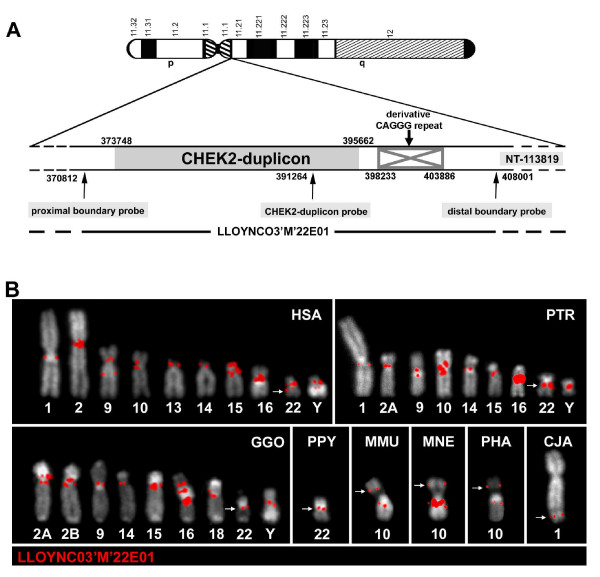
**FISH with a *CHEK2 *duplicon containing Y-chromosomal cosmid probe in anthropoids**. (A): Schematic representation of the human Y chromosome with an enlarged view of the *CHEK2 *duplicon in Yq11.1/Yq11.21. Basepair positions within the Y-chromosomal contig NT_113819 are indicated for the *CHEK2 *duplicon boundaries, for all probes used to identify a *CHEK2 *duplicon containing cosmid and for the derivative CAGGG repeat sequences. Localization of FISH probe LLOYNC03'M'22E01 within the Y-chromosomal contig is shown below. (B) Comparative FISH of cosmid LLOYNC03'M'22E01 (labelled in red) covering the human Y-chromosomal derivative *CHEK2 *duplicon in Yq11.1/Yq11.21 on human (HSA), chimpanzee (PTR), gorilla (GGO), orangutan (PPY), rhesus macaque (MMU), pig-tailed macaque (MNE), baboon (PHA) and marmoset (CJA) metaphase chromosomes. The great ape chromosomal designations are given according to [65], for MMU, MNE and PHA according to [47] and for CJA according to [66]. White arrows indicate the ancestral *CHEK2 *locus.

To investigate the evolution of the *CHEK2 *duplicon we screened a Y-chromosomal cosmid library (LLOYNC03"M") with a Y-derived *CHEK2 *duplicon probe (for detail see Methods). A total of 13 positive clones were detected. Probes bordering the Y-chromosomal *CHEK2 *duplicon were subsequently used to identify cosmids containing the entire derivative duplicon. Out of four positive clones cosmid LLOYNC03"M"22E01 (Figure 1A) was chosen for comparative FISH on human, great ape, Old World monkey (OWM) and New World monkey (NWM) metaphase chromosomes (Figure 1B, Table 1). In the human genome cosmid 22E01 hybridized to euchromatin/heterochromatin transition regions Yq11.1/Yq11.21 and Yq11.23/Yq12, and to additional 12 transition regions on 9 different autosomes. The only interstitial hybridization signal detected was assigned to the ancestral *CHEK2 *locus in 22q12.1. With only a few exceptions signals were detected in orthologous positions on chimpanzee and gorilla chromosomes (Figure 1B, Table 1). Signals on human and chimpanzee chromosomes 1 and 10 were missing on the orthologous gorilla chromosomes and species-specific signals were restricted to human chromosome 13, and to gorilla chromosomes 2B and 18. In contrast to the multi-site signal patterns found in all African great ape species, in the genomes of orangutan, the rhesus macaque and the common marmoset signals were detected at chromosomal sites orthologous to human 22q12.1. Additional signals with cosmid 22E01 were only detected in the proximal long arm (10qprox) on the NOR (nucleolus organizer region) – containing "marker-chromosome" 10 [47] of all three investigated OWM species (rhesus macaque, pig-tailed macaque and baboon). Sequences covered by cosmid 22E01 were subjected to repeat finding programs (for detail see Methods). These analyses suggested that the additional signal on the repeat-rich OWM "marker-chromosome" may be due to diverged CAGGG repeats within the Y-chromosomal cosmid probe. Such CAGGG repeats were previously identified as a single repeat block on the proximal long arm of the "marker-chromosome" of the crab-eating macaque [12] and were shown to have been distributed toward many pericentromeric regions during great ape evolution [12,48]. In addition, preliminary analysis of other Y-chromosomal SDs indicate that there may be further diverged CAGGG repeat sequences located on the Y chromosome that remained undetected by repeat finding programs. These undetected CAGGG repeat sequences may be located in the Yq11.23/Yq12 transition region and in the proximal part of the Yq11.1/Yq11.21 transition region. FISH with large insert genomic clones from both regions showed signals on the proximal long arm of the OWM chromosome 10 [25].

**Table 1:** Comparative FISH results of cosmid clone LLNLYC03"M" 22E01 from Yq11.1/q11.21

HSA	PTR	GGO	PPY	MMU	MNE	PHA	CJA
1q12/q21	1q12/q21						
2p11.1		2Aq11.1					
2q11.1	2Ap11.1	2Ap11.1 2Bp11.1 2Bq11.1					
9p11.1 9q12/q13	9p11 9q12/q13	9p11					
10p11.1/p11.2 10q11.2	10p11.1/p11.2 10q11.2						
13p11.2							
14p11.2	14p11	14p11					
15p11.2 15q11.2	15p11	15p11					
16p11.1/p11.2	16p11.1-16q11	16p11/p11.1 16p11.1/p11.2 16p12					
		18cen					
				10qprox	10qprox	10qprox	
22q11.1 22q12	22q11 22q12	22q12	22q12	10pprox (22q12)	10pprox (22q12)	10pprox (22q12)	1qdist (22q12)
Yq11.1/q11.21 Yq11.23/q12	Yp11.2	Yq11.2					

FISH results of the cosmid clone LLNLYC03"M"22E01 located in Yq11.1/Yq11.21 are listed for HSA (*H. sapiens*), PTR (*P. troglodytes*), GGO (*G. gorilla*), PPY (*P.pygmaeus pygmaeus*), MMU (*M. mulatta*), MNE (*M. nemestrina*), PHA (*P. hamadryas*) and CJA (*C.jacchus*). The chromosomal designations for the great apes are given according to [65], for MMU, MNE and PHA according to [47] and for CJA according to [66]. The human orthologous regions for the latter five species are indicated in parentheses. For PTR, GGO and PPY chromosome 16 banding nomenclature see Additional File 1.

### Comparative FISH with *CHEK2 *fosmid probes from 22q12.1 in anthropoids

**Figure 2:**
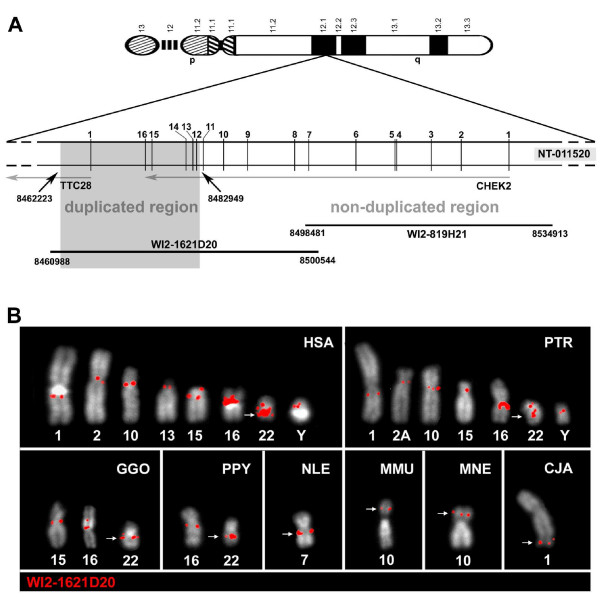
**FISH with a *CHEK2 *fosmid probe from 22q12.1 in anthropoids**. (A): Schematic representation of human chromosome 22 with an enlarged view of the analysed *CHEK2*/*TTC28*- locus in 22q12.1. All *CHEK2 *exons and exon 1 of *TTC28 *are given. Basepair positions for the duplicated region within the chromosome 22 contig NT_011520 are indicated. Localisations of fosmids WI2-1621D20, covering the duplicated region, and WI2-819H21, covering the *CHEK2 *non-duplicated region are given below. (B) Comparative FISH of fosmid WI2-1621D20 (labelled in red) covering the ancestral duplicated *CHEK2 *locus in 22q12.1 on human (HSA), chimpanzee (PTR), gorilla (GGO), orangutan (PPY), white-cheeked crested gibbon (NLE), rhesus macaque (MMU), pig-tailed macaque (MNE) and marmoset (CJA) metaphase chromosomes. The great ape chromosomal designations are given according to [65], for NLE according to [67], for MMU, MNE and PHA according to [47] and for CJA according to [66]. White arrows indicate the ancestral *CHEK2 *locus.

For unambiguous detection of *CHEK2 *duplicons in anthropoids we performed further FISH experiments with fosmid WI2-1621D20 (G248P81803F6; Figure 2A), which derives from human chromosome 22q12.1. This fosmid contains the complete duplicated portion of the ancestral *CHEK2*/*TTC28 *locus. No CAGGG repeat sequences are contained within this *CHEK2 *duplicon probe. Apart from the signal tagging the ancestral *CHEK2 *locus in 22q12.1 the probe showed the expected multi-site signal pattern in human (Figure 2B, Table 2). Signals were detected on 8 different chromosomes. On chromosomes 1, 2, 10, 13 and Y signals seemed to be restricted to one chromosomal location, whereas chromosomes 15, 16 and 22 showed a signal cluster, indicating the presence of at least two *CHEK2 *duplicons on each of these three chromosomes. In contrast to the Y-chromosomal cosmid probe no signals were detected with the fosmid on human chromosomes 9, 14 and Yq11.23/Yq12 (Table 1, Table 2; Figure 1B, Figure 2B). This observation might be explained by the absence of CAGGG repeat sequences in the fosmid probe. The fosmid WI2-1621D20 hybridized to 6 different autosomal chimpanzee chromosomes and the observed signal distribution was highly similar to the human pattern. Nevertheless, no signal was detected on chimpanzee chromosome 13 and only a single signal on chimpanzee chromosome 15. Substantial differences to the Y-chromosomal cosmid probe were detected in the gorilla genome. Only three gorilla chromosomes were tagged. Gorilla chromosome 16 showed two signals located in the proximal and distal transition regions of the heterochromatin block on the short arm (Additional File 1), indicating gorilla-specific reorganization or acquisition of *CHEK2 *duplicons on this chromosome. Gorilla chromosome 22 showed two distinct signal localizations. One signal mapped to the ancestral interstitial *CHEK2 *locus and one to the pericentromeric region of the long arm. As in the chimpanzee genome, this probe generated no signal on gorilla chromosome 13, favouring a human-specific *CHEK2 *duplication event towards the short arm of human chromosome 13. The orangutan genome revealed differences in the signal pattern between both probes, too. In contrast to the Y-chromosomal cosmid probe, two signals were detected on orangutan chromosomes 16 and 22. This observation indicates the occurrence of a first *CHEK2 *duplication event before the radiation of the great apes and, in addition, shows that sequences located on chromosome 16 are more related to the ancestral than to the derivative locus on the human Y chromosome. Furthermore, hybridization of fosmid WI2-1621D20 on metaphase chromosomes of the white-cheeked crested gibbon, the rhesus macaque, the pig-tailed macaque and the common marmoset revealed a single *CHEK2 *copy status. In all these species, only the chromosomal region orthologous to human chromosome 22q12 was labelled. This observation contrasts the signal location of the Y-chromosomal cosmid probe in the analysed OWM species, but can again be explained by the absence of CAGGG repeat sequences in the ancestral *CHEK2 *fosmid probe.

**Table 2:** Comparative FISH results of fosmid clone WI2-1621D20 from 22q12.1

HSA	PTR	GGO	PPY	NLE	MMU	MNE	CJA
1q12/q21	1q12/q21						
2p11.1							
2q11.1	2Ap11.1						
10p11.1/p11.2	10p11.1/p11.2						
13p11.2							
15p11.2 15q11.2	15p11	15p11					
16p11.1/p11.2	16q11/q12.1	16p11/p11.1 16p11.1/p11.2	16q11/q12.1				
22q11.1 22q12	22q11 22q12	22q11 22q12	22q12	7qdist (22q12)	10pprox (22q12)	10pprox (22q12)	1qdist (22q12)
Yq11.1/q11.21	Yp11.2						

FISH results of the fosmid clone WI2-1621D20 located in 22q12.1 are listed for HSA (*H. sapiens*), PTR (*P. troglodytes*), GGO (*G. gorilla*), PPY (*P.pygmaeus pygmaeus*), NLE (*N. leucogenys*) MMU (*M. mulatta*), MNE (*M. nemestrina*) and CJA (*C.jacchus*). The chromosomal designations for the great apes are given according to [65], for NLE according to [67], for MMU and MNE according to [47] and for CJA according to [66]. The human orthologous regions for the latter five species are indicated in parentheses. For PTR, GGO and PPY chromosome 16 banding nomenclature see Additional File 1.

To verify the single-copy status of the non-duplicated portion of the *CHEK2 *locus, we performed FISH with the fosmid probe WI2-819H21 (G248P81285D11; Figure 2A). In all analysed primate species, including human, this probe hybridized to a single genomic localisation corresponding to the human 22q12.1 orthologous regions. In conclusion, our comparative FISH results with fosmid probe WI2-1621D20 (Figure 2B, Table 2) show the single-copy status of *CHEK2 *in all tested New World and Old World monkeys and in the white-cheeked crested gibbon. While a first *CHEK2 *duplication event was detected in the orangutan, a burst of duplication, giving rise to the complex signal pattern, occurred before the radiation of African great apes. Except for chromosomes 16 and Y all signals present in non-human primates were also detected in the orthologous human regions.

### Comparative in silico analysis of *CHEK2 *duplicons in anthropoids

**Figure 3:**
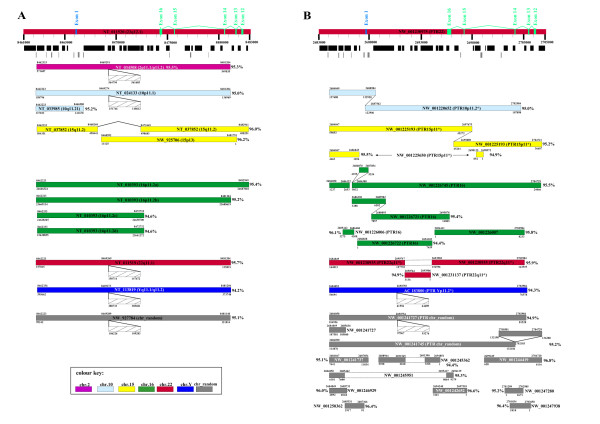
**Comparative *in silico *analysis of *CHEK2 *duplicons in human and chimpanzee**. Genomic structure of the *CHEK2 *duplicon family in the (A) human (NCBI Build36.2) and (B) chimpanzee (NCBI Build2) sequence assembly. The respective ancestral *CHEK2 *locus is depicted by the top horizontal red bar. Within each bar the corresponding accession numbers of the contigs and the position of the *CHEK2 *exons (green rectangles) and *TTC28 *exon (blue rectangle) are given. The precise genomic extension of the ancestral loci within each contig is shown beneath. The next two layers illustrate the common repeat and simple repeat content of the ancestral loci as black and grey boxes, respectively. All derivative duplicons are shown below using the indicated colour key. Precise positions of each derivative duplicon within the corresponding contig and the genomic extension of the sequence homology shared with the ancestral locus are indicated below and above each bar, respectively. Striped boxes represent LINE1 elements. LINE1 element integration-sites within the respective contigs are given below and the position of homology break within the ancestral locus is given above. Overall sequence identities between the paralogous human and chimpanzee *CHEK2 *duplicons and the respective ancestral *CHEK2 *sequences are given next to all *CHEK2 *duplicons.

We used the sequence from the human ancestral *CHEK2 *locus for megaBLAST analysis (Basic Local Alignment Search Tool; http://www.ncbi.nlm.nih.gov/blast/Blast.cgi) of the human genome. This analysis revealed the presence of 10 derivative *CHEK2 *duplicons in the current human reference genome assembly, while two additional duplicons were identified in the Celera whole genome assembly (Figure 3A). The largest *CHEK2 *duplicon is located in 16p11.2 (16p11.2a) and spans 20747 basepairs of the ancestral *CHEK2 *locus. This duplicon includes exons 12 to 16 of *CHEK2 *and exon 1 of *TTC28*. Furthermore, three additional smaller duplicons (16p11.2b-d) were detected on chromosome 16. Duplicons 16p11.2c and 16p11.2d are about 10 kb in size and share 99% sequence identity, indicating a recent duplication event. In addition, both duplicons terminate within an AluSq-element, which has been shown to be enriched within SD junctions [11]. Slightly shorter than duplicon 16p11.2a is the *CHEK2 *duplicon located in 15q11.2. A second *CHEK2 *duplicon on chromosome 15 was only detected in the Celera whole genome assembly and is assigned to chromosome band 15p13. Both chromosome 15 duplicons share 99% sequence identity. As our detailed FISH analysis also indicated the presence of at least two different *CHEK2 *duplicon copies on human chromosome 15, we conclude that these two duplicons are the result of a recent intrachromosomal duplication event or might reflect individual genomic variation. Furthermore, the 15q11.2 duplicon exhibits an internal 4.2 kb deletion not present in the 15p13 duplicon. Both deletion breakpoints reside in AluSq-elements, one of them being identical to the one terminating the duplicons 16p11.2c and 16p11.2d. This deletion was shown to be present in two different BAC libraries, RPCI-11 and RPCI-13, thus almost certainly ruling out individual variation. In summary, duplicons located at chromosomes 15 and 16 revealed a high intrachromosomal sequence identity. This observation is concordant with previous investigations showing that intrachromosomal duplications share higher sequence identity than interchromosomal duplications, thus pointing to a recent intrachromosomal expansion of the human genome [16,49]. It should be mentioned that the high degree of sequence identity between intrachromosomal duplicons might also be due to nonallelic homologous recombination (NAHR) and/or gene conversion [50-53]. Additional *CHEK2 *duplicons, containing almost the entire *CHEK2 *duplicated portion, are located in 2p11.1/2p11.2, 10p11.1, 22q11.1 and Yq11.1/Yq11.21. Furthermore, one *CHEK2 *duplicon sequence in the human genome remains chromosomally unassigned (Chr_random), but may be located in the 1q12/1q21 euchromatin/heterochromatin transition region or on the short arm of chromosome 13. FISH signals within these two regions (Figure 2B) do not correspond to any chromosomally assigned *CHEK2 *duplicon in the current human whole genome assemblies. Interestingly, duplicons located in 2p11.1/2p11.2, 10p11.1, 22q11.1, Yq11.1/Yq11.21 and Chr_random share the same 3 kb LINE1 (long interspersed nuclear element 1) integration, thereby suggesting a common origin. The smallest *CHEK2 *duplicon was found in 10q11.21 and is about 4.6 kb in size.

To elucidate the molecular evolution of the derivative *CHEK2 *duplicons we investigated their flanking paralogous sequences. All known *CHEK2 *duplicons extend into the same proximal duplicated sequence, indicating a single initial pericentromeric "seeding" event. Subsequent pericentromeric "swaps" of the larger duplicon cassette led to the *CHEK2 *duplicon distribution observed in the human genome. The ancestral location of the duplicon proximal to all *CHEK2 *duplicons resides within the *IGL@ *locus in 22q11.21. The duplicated sequences extending beyond the distal *CHEK2 *duplicon junctions are not identical for all derivative duplicons. While *CHEK2 *duplicons 16p11.2a and 15q11.2 show the same distal homology, duplicons 2q11.1/2q11.2, 10p11.1, 16p11.2b, 22q11.1, Yq11.1/Yq11.21 and Chr_random share a different distal duplicated sequence. Thus, the latter set of duplicons may have arisen by a duplication of the 16p11.2b duplicon, followed by a LINE1 element integration and successive pericentromeric exchange.

Subsequently, we used the same *in silico *approach to determine the *CHEK2 *duplicon architecture of the chimpanzee genome (build2). Chromosomally assigned duplicons in the chimpanzee genome showed chromosomal designations corresponding to the human genome locations. Duplicons were detected on chimpanzee chromosomes 10, 15, 16, 22 and Y (Figure 3B), but not on chromosome 2A. Two duplications containing almost the entire *CHEK2 *duplicon and 11 smaller duplicon fragments were not chromosomally assigned in the current chimpanzee whole genome assembly. Similar to the human genome the largest *CHEK2 *duplicon is assigned to chromosome 16 and a slightly shorter *CHEK2 *duplicon is located on chromosome 15. In contrast to the human genome the chromosome 15 duplicon contains no deletion, indicating that this deletion is human specific. In addition, duplicons on chimpanzee chromosomes 10 and 15 contained small internal duplications and chromosome 16 duplicons seemed to be more fragmented (Figure 3B). These findings might be explained by chimpanzee-specific rearrangements or, more likely, by the inherent problem of generating highly reliable contiguous sequence assemblies in regions enriched in SDs.

Comparative *in silico *analysis (BLAT search http://genome.ucsc.edu/ of the orangutan (ponAbe2) and rhesus macaque (rheMac2) whole genome assemblies using the human duplicated *CHEK2 *sequence yield just one copy in the respective genomes on their orthologous chromosome 22. No *CHEK2 *duplicon was found on orangutan chromosome 16 by this approach, but this may be due to the underrepresentation of segmentally duplicated sequences within pericentromeric regions in the whole genome assemblies [49]. The unduplicated status of *CHEK2 *in the rhesus macaque genome assembly is concordant with our FISH results obtained with fosmid WI2-1621D20.

### Phylogenetic reconstruction of *CHEK2 *duplicon events in anthropoids

**Figure 4:**
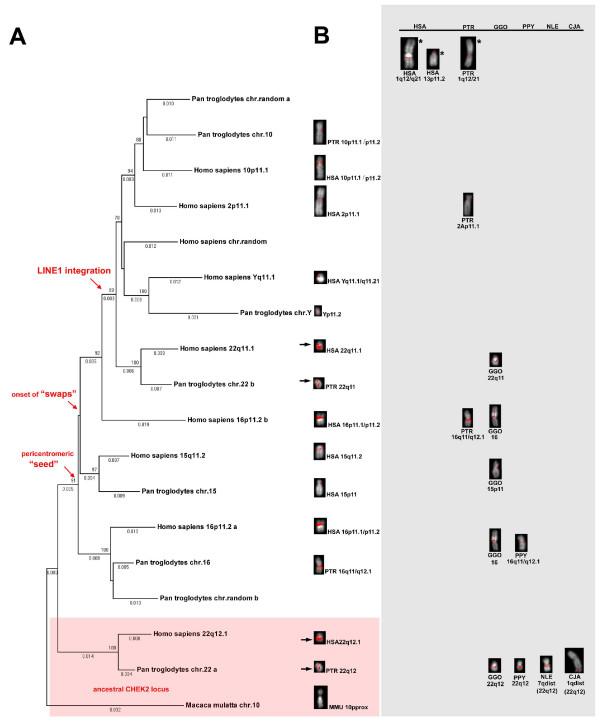
**Phylogenetic reconstruction of *CHEK2 *duplicon events in anthropoids**. (A) Phylogenetic analysis of the *CHEK2 *duplicon family. A neighbor-joining phylogram was generated using MEGA 4.0 software [41] and rooted on the ancestral *CHEK2 *sequence of the rhesus macaque. We used 1951 bp of human and chimpanzee *CHEK2 *duplicons and ancestral *CHEK2 *sequences of human, chimpanzee and rhesus macaque. Ancestral loci are highlighted by a red box. Branch lengths are proportional to the number of nucleotide changes between taxa and are indicated below each respective branch (>0.001). Bootstrap values > 70 from 1000 replicates are indicated above each corresponding branch point. Red arrows indicate the LINE1 integration and the pericentromeric "seed" and onset of "swaps". (B) Corresponding FISH signals with fosmid WI2-1621D20 on chromosomes of human (HSA), chimpanzee (PTR), gorilla (GGO), orangutan (PPY), white-cheeked crested gibbon (NLE), rhesus macaque (MMU) and common marmoset (CJA). The left column represents all chromosomes, for which signals were unambiguously correlated to the respective sequences used for MEGA 4.0 analysis. Black arrows indicate the corresponding FISH signals on chromosomes 22. All other signal-containing chromosomes for which no corresponding sequence was used for MEGA4 analysis are arranged in the grey box. These chromosomes are ordered with respect to their human orthologous chromosomes. Chromosomes showing a signal for which no corresponding *CHEK2 *duplicon sequence was available are displayed on the top and are indicated by asterisks.

To further delineate the evolution of the *CHEK2 *duplicons we performed phylogenetic analysis with non-coding and non-repetitive sequences using the MEGA4.0 [41] software (Figure 4A). The multi-sequence alignment was composed of 1951 basepairs derived from two loci within the *CHEK2 *duplicons. The proximal sequence is located upstream of exon 1 of *TTC28 *(NT_011520: 8465388–8465900) and the distal sequence is located in intron 14 of *CHEK2 *(NT_011520: 8478430–8480219). We aligned all human and chimpanzee derivative duplicons as well as the ancestral sequences of human, chimpanzee and rhesus macaque. Our phylogenetic analyses placed all chromosomally assigned chimpanzee *CHEK2 *duplicons next to the human *CHEK2 *duplicons showing the same chromosomal designations (Figure 4A). We therefore concluded the orthologous nature of the particular human and chimpanzee *CHEK2 *duplicons on chromosomes 10, 15, 16, 22 and Y. Based on an estimated divergence time of 25 million years between human and rhesus macaque lineages [43], we calculated the effective nucleotide substitution rates (r = K/2 T) (Additional File 2). Calculated times for pericentromeric "seed" and onset of "swaps", are ~22.3 MYA and ~15.9 MYA, respectively. Replacing the human lineage by the chimpanzee lineage, a pericentromeric seed of ~21.2 MYA and an onset of swaps of ~18.0 MYA was calculated. Both independent calculations placed the initial duplication event before the radiation of orangutan and African great apes about 15 MYA [43] thereby coinciding with our FISH results of fosmid clone WI2-1621D20 on orangutan metaphase chromosomes (Table 2; Figure 2B). No signs of duplication were detected on metaphase chromosomes of the white-cheeked crested gibbon, which is thought to have split from the great ape-human lineage about 18 MYA. Only one signal in 7qdist representing the orthologous region of human 22q12 was detected by FISH (Table 2; Figure 2B). This might either be explained by the loss of the primary *CHEK2 *duplicon in the gibbon lineage or the high degree of sequence divergence between the putative gibbon duplicon and the human ancestral *CHEK2 *locus. Such a high degree of sequence divergence is a very likely explanation supported by previous investigations, which detected an acceleration of substitution rates after the duplication event [54-56]. Phylogenetic reconstruction of the duplication events revealed that the largest human and chimpanzee duplicons located on chromosome 16 share the highest degree of sequence identity with the ancestral *CHEK2 *locus (Additional File 3). This indicates, in combination with our FISH results, that the first duplication event was a pericentromeric "seed" from the ancestral interstitial *CHEK2 *locus toward the pericentromeric region of chromosome 16 (Figure 4B). The duplicons located on chromosomes 15 of chimpanzee and human share the highest degree of sequence identity with the largest chromosome 16 duplicons of human and chimpanzee (Additional File 3) suggesting a pericentromeric "swapping" event from chromosome 16 towards chromosome 15. Phylogenetic reconstruction identifies the duplicons located in the pericentromeric regions of human chromosome 2 and human and chimpanzee chromosomes 10, 22 and Y as the most recent ones in the respective genomes. This finding concurs with the *in silico *results, which showed these duplicons to harbour a unique LINE1 element integration.

### Detection of inter-species copy number variations of *CHEK2 *by quantitative PCR

**Figure 5:**
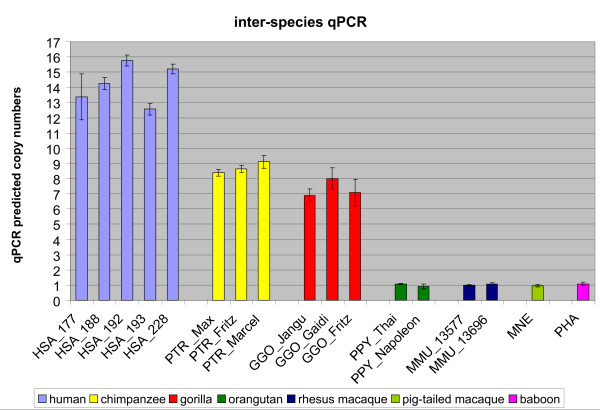
***CHEK2 *copy number prediction by inter-species quantitative PCR**. Human (HSA, blue), chimpanzee (PTR, yellow), gorilla (GGO, red), orangutan (PPY, green), rhesus macaque (MMU, dark blue), pig-tailed macaque (MNE, light green) and baboon (PHA, purple) individuals were analysed. Calculated standard errors for each sample are indicated. Genomic DNA of the rhesus macaque MMU#13577 was used for standard curve construction.

The paralogous *CHEK2 *duplicons in human and African great apes are part of SDs. SDs are known to be frequently associated with copy number variation [57-60]. A robust approach to specifically target such variable regions is quantitative PCR (qPCR). To independently determine the *CHEK2 *duplicon numbers in the genomes of anthropoids we performed inter-species quantitative PCR with primers located in exon 14. The complete alignment of exonic sequences from all known *CHEK2 *duplicons of the human, chimpanzee, orangutan and rhesus macaque genome was used to determine the set of primers best matching the sequences. Nevertheless, we cannot rule out that these primers do not match perfectly to all *CHEK2 *duplicons in all analysed species. As FISH and *in silico *analyses consistently demonstrated the single copy status of *CHEK2 *in the rhesus macaque genome we used rhesus macaque genomic DNA as the reference DNA for *CHEK2 *copy number prediction in human, great ape and OWM species. We determined the copy numbers of five different human individuals, three chimpanzee and gorilla individuals each, two orangutan and rhesus macaque individuals each and one individual each of the pig-tailed macaque and baboon (Figure 5; Additional File 4). All analysed baboon, pig-tailed macaque, rhesus macaque and orangutan individuals revealed just one *CHEK2 *copy per haploid genome. A drastic increase in copy number was detected for the African great apes with seven and nine *CHEK2 *copies in the haploid genomes of gorilla and chimpanzee, respectively. The human genome presented the highest increase in *CHEK2 *copy number, with a variation between 13 to 16 *CHEK2 *copies per haploid human genome. Our results significantly demonstrate, that the analysed human individuals have a higher *CHEK2 *copy number than chimpanzee and gorilla (p < 0.0001) and a higher *CHEK2 *copy number than orangutan and all analysed OWMs (p < 0.0001). In addition, chimpanzee and gorilla have a significantly higher *CHEK2 *copy number than orangutan and OWMs (p < 0.0001). This observation confirms our FISH investigation and phylogenetic analysis, which placed the burst of *CHEK2 *duplication after the radiation of orangutan and African great apes. Furthermore, we detected different numbers of *CHEK2 *copies within the analysed human individuals. Taking into account the sample size, we can only presume whether there is *CHEK2 *copy number variation in the human population. As mentioned above, our qPCR approach revealed only one *CHEK2 *copy per haploid orangutan genome, whereas FISH detected two signals on orangutan chromosomes 16 and 22 (Figure 2B) and phylogenetic analyses indicated the occurrence of a first *CHEK2 *duplication event before the radiation of orangutan and African great apes (Figure 4). The failure to amplify the second *CHEK2 *copy in the orangutan genome might be explained either by primer mismatch or loss of exon 14 in the orangutan chromosome 16 copy. Therefore, real *CHEK2 *copy numbers in non-human primates may be even higher than were predicted by our approach.

## Conclusion

*CHEK2*, which is essential for genomic stability [27], is known to be a multiorgan cancer susceptibility gene and is frequently analysed in tumour diagnostics, e.g. of breast, colorectal and prostate cancer [61,62]. *CHEK2 *is one of a multitude of genes known to be part of SDs [10,63]. Our detailed three-pronged approach clearly demonstrates that *CHEK2 *duplicons show a high degree of both copy number variation and sequence identity. Furthermore, there is strong evidence, that not all duplicons in the human genome have been sequenced yet. Thus, molecular diagnostics of *CHEK2 *inside the duplicated region might be hampered by the individual-specific set of *CHEK2 *duplicons. One previously published variant (1422delT) of the functional *CHEK2 *gene, was thought to predispose to Li-Fraumeni syndrome [64], but subsequently was shown to be the genomic sequence of a *CHEK2 *duplicon [63]. To avoid such diagnostic pitfalls in the analysis of duplicated disease related genes, it is essential to close the still existing gaps in the human genome sequence by closely examining segmentally duplicated regions. Additionally, copy number and sequence variation within SDs might require further efforts to adapt the diagnostic settings to different ethnic backgrounds.

Furthermore, our detailed *CHEK2 *analysis revealed its highly dynamic nature during anthropoid evolution. Both, FISH and phylogenetic analyses suggest the first duplication event to have occurred before the radiation of the great ape species. Extensive pericentromeric exchange and intrachromosomal duplication events led to a burst of *CHEK2 *duplications before the radiation of the African great apes followed by lineage specific rearrangements creating species-specific distribution patterns in great apes and human. In conclusion, our analysis of the *CHEK2 *duplicon evolution reveals, that SDs contribute to inter-species variation.

## Authors' contributions

CM designed as well as carried out the experiments of the study and drafted the manuscript. AMGF performed the statistical analyses and helped in finalising the corresponding sections of the manuscript. SK and WS participated in the design of the study and helped to finalise the manuscript. All authors proofread and approved the final manuscript.

## Supplementary Material

Additional file 1 **Chromosome 16 banding nomenclature and signal localization of clone WI2-1621D20 in anthropoids. **Indicated are the FISH signal localizations of clone WI2-1621D20 (red) on chromosome 16 in great apes and human. Human chromosome 16 nomenclature according to ISCN (2005). Chromosome 16 banding pattern of great apes (PTR, GGO and PPY) according to Goidts et al. (2005). Chromosome 16 banding nomenclature of great apes according to ISCN (1985) and adjusted to the human chromosome 16 banding nomenclature. Black bars represent the evolutionary breakpoints as described by Goidts et al. (2005). References: ISCN (2005): **An international system for human cytogenetic nomenclature**. Shaffer LG, Tommerup N (eds): S. Karger, Basel 2005. ISCN (1985): **An international system for human cytogenetic nomenclature**. Harnden DG, Klinger HP (eds): S. Karger, Basel 1985. Goitds V, Szamalek JM, de Jong PJ, Cooper DN, Chuzhanova N, Hameister H, Kehrer-Sawatzki H.: **Independent intrachromosomal recombination events underlie the pericentromeric inversion of chimpanzee and gorilla chromosomes homologous to human chromosome **16, *Genome Res *2005, **15**(9): 1232–42 Click here for file

Additional file 2 **Evolutionary distances overview.** Based on the Kimura two-parameter model the average number of nucleotide substitutions per site (K) were calculated for outgroup distances: all human sequences to macaque sequences (HSA to MMU), all human non-ancestral paralogs to the macaque outgroup (MMU to Para) and the human ancestral duplicon to the macaque outgroup (MMU to Anc). Comparison of these values with two human interparalog distances, i.e. human ancestral to all human paralogs (Anc to Para) and the average K of all human paralogs (all Para), provides information on the timing of the initial duplicationevent (Macaque Seed) and the onset of secondary duplications (Macaque Swaps). We calculated locus-specific effective nucleotide substitution rates (r = K/2 T) based on an estimated divergence time of 25 million years between the human and the Old World monkeys. In addition, all calculations were performed replacing the human lineage by the chimpanzee lineage. Click here for file

Additional file 3 **Sequence Identity Matrix of sequences used for the phylogenetic analysis.** We created a sequence identity matrix of all sequences, which were used for our phylogenetic analysis. Multi-sequence alignments were composed of 1951 basepairs derived from two loci within the *CHEK2 *duplicons. The proximal sequence is located upstream of exon 1 of *TTC28 *(NT_011520: 8465388–8465900) and the distal sequence is located within intron 14 of *CHEK2 *(NT_011520: 8478430–8480219). The sequence identity matrix of these sequence alignments was generated with the BioEdit software (version 7.0.0). Click here for file

Additional file 4 **qPCR predicted CN calculations.** qPCR reactions were performed in duplicate. Copy number mean, standard deviation (SD) and standard error (SE) were calculated for all analysed human (HSA), chimpanzee (PTR), gorilla (GGO), orangutan (PPY), rhesus macaque (MMU), pig-tailed macaque (MNE) and baboon (PHA) normalised samples. Standard errors of the normalised *CHEK2 *copy numbers were calculated from the standard deviations of the values of the *CFTR *and *CHEK2 *genes using the formula provided by the user menu (ABI Prism 7700 Sequence Detection System, User Bulletin no.2 1997, p.34). P-values were calculated by a 2-tailed Student t-test. Click here for file
