# Supplementary material for: Evolutionary analysis of the highly dynamic CHEK2 duplicon in anthropoids
Source: BMC Evol Biol. 2008 Oct 2;8:269. doi: 10.1186/1471-2148-8-269 (PMC2566985; doi:10.1186/1471-2148-8-269)

Additional File 1:

Chromosome 16 banding nomenclature and signal localization of clone WI2-1621D20 on anthropoid chromosomes

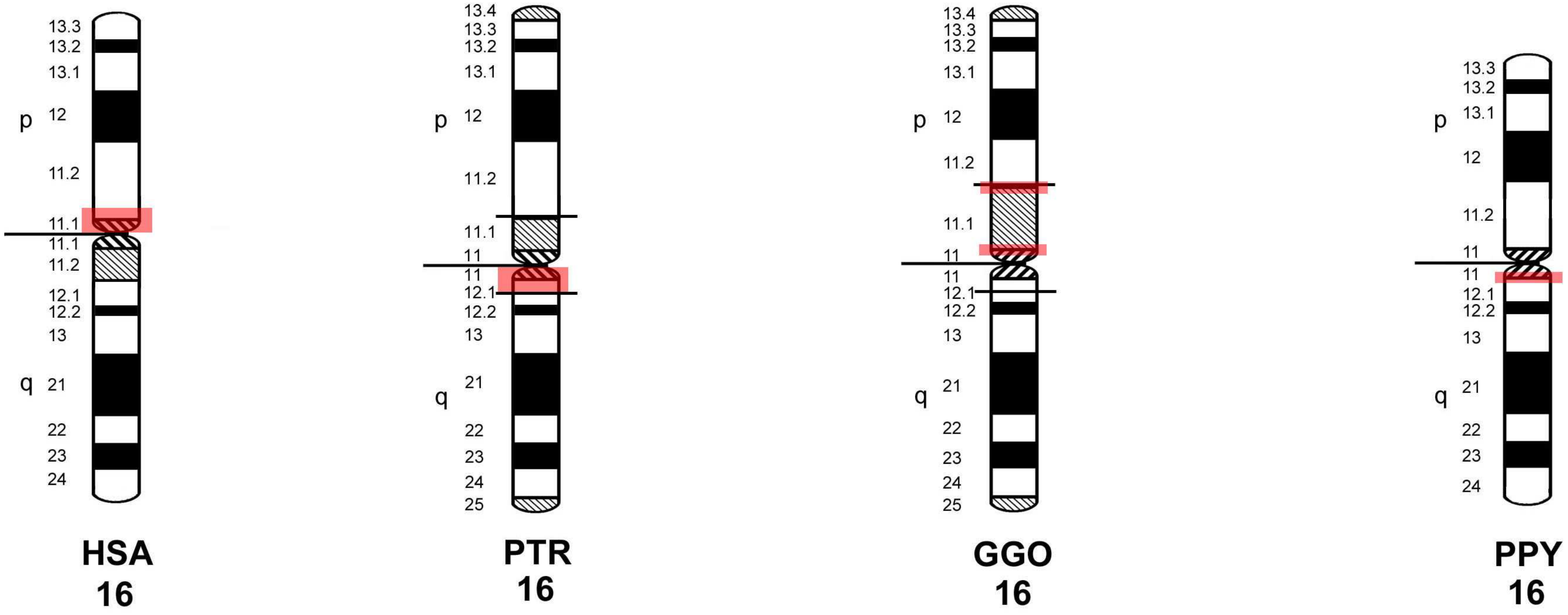

Supplement: Additional file 1 — Chromosome 16 banding nomenclature and signal localization of clone WI2-1621D20 in anthropoids. Indicated are the FISH signal localizations of clone WI2-1621D20 (red) on chromosome 16 in great apes and human. Human chromosome 16 nomenclature according to ISCN (2005). Chromosome 16 banding pattern of great apes (PTR, GGO and PPY) according to Goidts et al. (2005). Chromosome 16 banding nomenclature of great apes according to ISCN (1985) and adjusted to the human chromosome 16 banding nomenclature. Black bars represent the evolutionary breakpoints as described by Goidts et al. (2005). References: ISCN (2005): An international system for human cytogenetic nomenclature. Shaffer LG, Tommerup N (eds): S. Karger, Basel 2005. ISCN (1985): An international system for human cytogenetic nomenclature. Harnden DG, Klinger HP (eds): S. Karger, Basel 1985. Goitds V, Szamalek JM, de Jong PJ, Cooper DN, Chuzhanova N, Hameister H, Kehrer-Sawatzki H.: Independent intrachromosomal recombination events underlie the pericentromeric inversion of chimpanzee and gorilla chromosomes homologous to human chromosome 16, Genome Res 2005, 15(9): 1232–42 [file 1471-2148-8-269-S1.pdf]
