# Supplementary material for: Evolutionary analysis of the highly dynamic CHEK2 duplicon in anthropoids
Source: BMC Evol Biol. 2008 Oct 2;8:269. doi: 10.1186/1471-2148-8-269 (PMC2566985; doi:10.1186/1471-2148-8-269)
Supplement: Additional file 2 — Evolutionary distances overview. Based on the Kimura two-parameter model the average number of nucleotide substitutions per site (K) were calculated for outgroup distances: all human sequences to macaque sequences (HSA to MMU), all human non-ancestral paralogs to the macaque outgroup (MMU to Para) and the human ancestral duplicon to the macaque outgroup (MMU to Anc). Comparison of these values with two human interparalog distances, i.e. human ancestral to all human paralogs (Anc to Para) and the average K of all human paralogs (all Para), provides information on the timing of the initial duplicationevent (Macaque Seed) and the onset of secondary duplications (Macaque Swaps). We calculated locus-specific effective nucleotide substitution rates (r = K/2 T) based on an estimated divergence time of 25 million years between the human and the Old World monkeys. In addition, all calculations were performed replacing the human lineage by the chimpanzee lineage. [file 1471-2148-8-269-S2.pdf]

Additional File 2. Evolutionary distance overview

Outgroup distances			Human interparalog distances					
Mean K HSA to MMU	Mean K MMU to Para	Mean K MMU to Anc	Mean K Anc to Para	Mean K all Para	Mac (Myr)	Mac rate (x 10^{-9})	Macaque Seed (Myr)	Macaque Swaps (Myr)
0.060689	0.061305	0.05567	0.049641	0.035485	25	1.11	22.29	15.94

Outgroup distances			Chimpanzee interparalog distances					
Mean K PTR to MMU	Mean K MMU to Para	Mean K MMU to Anc	Mean K Anc to Para	Mean K all Para	Mac (Myr)	Mac rate (x 10^{-9})	Macaque Seed (Myr)	Macaque Swaps (Myr)
0.0606	0.061667	0.05313	0.04509	0.038185	25	1.06	21.22	17.97
