# Supplementary material for: Evolutionary analysis of the highly dynamic CHEK2 duplicon in anthropoids
Source: BMC Evol Biol. 2008 Oct 2;8:269. doi: 10.1186/1471-2148-8-269 (PMC2566985; doi:10.1186/1471-2148-8-269)
Supplement: Additional file 3 — Sequence Identity Matrix of sequences used for the phylogenetic analysis. We created a sequence identity matrix of all sequences, which were used for our phylogenetic analysis. Multi-sequence alignments were composed of 1951 basepairs derived from two loci within the CHEK2 duplicons. The proximal sequence is located upstream of exon 1 of TTC28 (NT_011520: 8465388–8465900) and the distal sequence is located within intron 14 of CHEK2 (NT_011520: 8478430–8480219). The sequence identity matrix of these sequence alignments was generated with the BioEdit software (version 7.0.0). [file 1471-2148-8-269-S3.pdf]

Additional File 3:

Sequence Identity Matrix of sequences used for the phylogenetic analysis

Seq->	HSA 22q12.1	PTR chr.22 a	HSA 15q11.2	HSA chr.random	HSA 22q11.1	HSA 16p11.2 a	HSA 16p11.2 b	HSA 10p11.1	HSA 2p11.1
HSA 22q12.1	ID	0,872	0,95	0,932	0,936	0,938	0,937	0,931	0,931
PTR chr.22 a	0,872	ID	0,851	0,832	0,839	0,836	0,834	0,831	0,833
HSA 15q11.2	0,95	0,851	ID	0,954	0,954	0,956	0,954	0,95	0,951
HSA chr.random	0,932	0,832	0,954	ID	0,965	0,948	0,956	0,965	0,962
HSA 22q11.1	0,936	0,839	0,954	0,965	ID	0,948	0,958	0,961	0,961
HSA 16p11.2 a	0,938	0,836	0,956	0,948	0,948	ID	0,96	0,944	0,947
HSA 16p11.2 b	0,937	0,834	0,954	0,956	0,958	0,96	ID	0,953	0,955
HSA 10p11.1	0,931	0,831	0,95	0,965	0,961	0,944	0,953	ID	0,966
HSA 2p11.1	0,931	0,833	0,951	0,962	0,961	0,947	0,955	0,966	ID
HSA Yq11.1	0,926	0,83	0,943	0,959	0,96	0,941	0,946	0,951	0,952
MMU	0,929	0,829	0,934	0,932	0,933	0,936	0,933	0,929	0,933
PTR chr.random a	0,93	0,833	0,948	0,959	0,964	0,946	0,955	0,97	0,965
PTR chr.16	0,944	0,843	0,963	0,953	0,955	0,982	0,964	0,95	0,952
PTR chr.15	0,945	0,844	0,974	0,953	0,954	0,957	0,954	0,951	0,951
PTR chr.10	0,934	0,832	0,953	0,963	0,959	0,948	0,958	0,966	0,963
PTR chr.random b	0,937	0,835	0,955	0,948	0,947	0,979	0,957	0,945	0,945
PTR chr.22 b	0,937	0,842	0,956	0,965	0,984	0,948	0,956	0,961	0,961
PTR chr.Y	0,918	0,823	0,938	0,951	0,951	0,929	0,937	0,945	0,944

HSA Yq11.1	MMU	PTR chr.random a	PTR chr.16	PTR chr.15	PTR chr.10	PTR chr.random b	PTR chr.22 b	PTR chr.Y
0,926	0,929	0,93	0,944	0,945	0,934	0,937	0,937	0,918
0,83	0,829	0,833	0,843	0,844	0,832	0,835	0,842	0,823
0,943	0,934	0,948	0,963	0,974	0,953	0,955	0,956	0,938
0,959	0,932	0,959	0,953	0,953	0,963	0,948	0,965	0,951
0,96	0,933	0,964	0,955	0,954	0,959	0,947	0,984	0,951
0,941	0,936	0,946	0,982	0,957	0,948	0,979	0,948	0,929
0,946	0,933	0,955	0,964	0,954	0,958	0,957	0,956	0,937
0,951	0,929	0,97	0,95	0,951	0,966	0,945	0,961	0,945
0,952	0,933	0,965	0,952	0,951	0,963	0,945	0,961	0,944
ID	0,92	0,953	0,944	0,943	0,95	0,938	0,96	0,963
0,92	ID	0,927	0,939	0,936	0,929	0,934	0,933	0,914
0,953	0,927	ID	0,953	0,948	0,967	0,947	0,963	0,947
0,944	0,939	0,953	ID	0,961	0,954	0,982	0,955	0,936
0,943	0,936	0,948	0,961	ID	0,953	0,957	0,956	0,936
0,95	0,929	0,967	0,954	0,953	ID	0,951	0,96	0,943
0,938	0,934	0,947	0,982	0,957	0,951	ID	0,947	0,928
0,96	0,933	0,963	0,955	0,956	0,96	0,947	ID	0,952
0,963	0,914	0,947	0,936	0,936	0,943	0,928	0,952	ID
