# Supplementary material for: Evolutionary analysis of the highly dynamic CHEK2 duplicon in anthropoids
Source: BMC Evol Biol. 2008 Oct 2;8:269. doi: 10.1186/1471-2148-8-269 (PMC2566985; doi:10.1186/1471-2148-8-269)
Supplement: Additional file 4 — qPCR predicted CN calculations. qPCR reactions were performed in duplicate. Copy number mean, standard deviation (SD) and standard error (SE) were calculated for all analysed human (HSA), chimpanzee (PTR), gorilla (GGO), orangutan (PPY), rhesus macaque (MMU), pig-tailed macaque (MNE) and baboon (PHA) normalised samples. Standard errors of the normalised CHEK2 copy numbers were calculated from the standard deviations of the values of the CFTR and CHEK2 genes using the formula provided by the user menu (ABI Prism 7700 Sequence Detection System, User Bulletin no.2 1997, p.34). P-values were calculated by a 2-tailed Student t-test. [file 1471-2148-8-269-S4.pdf]

Additional file 4:

qPCR predicted CN calculations

	Mean CN Chek 2	Mean CN CFTR	SD Chek2	SD CFTR	CV Chek2	CV CFTR	Normalized CN	SD	SE
HSA_177	48,923626	3,6567507	0,006160858	0,584709	0,000125928	0,159898513	13,37898862	2,139281052	1,512700138
HSA_188	44,249054	3,1009426	1,4626211	0,06699848	0,033054291	0,021605843	14,26954952	0,563493401	0,398450005
HSA_192	74,87987	4,7549477	1,2357267	0,13143209	0,016502789	0,027641122	15,74777994	0,506964471	0,358478015
HSA_193	47,48066	3,7764034	0,8188627	0,14370322	0,017246237	0,038052932	12,57298413	0,525282707	0,371430964
HSA_228	72,51634	4,7667284	1,771635	0,08229594	0,024430839	0,017264659	15,21302116	0,455104418	0,32180742
PTR _Max	48,943977	5,8306694	1,0816054	0,17056863	0,022098846	0,029253696	8,394229486	0,307753302	0,217614447
PTR_Fritz	42,43753	4,9129114	1,2546922	0,103059836	0,029565627	0,020977345	8,637959561	0,313139475	0,221423046
PTR_Marcel	49,725464	5,460699	0,72260064	0,34492508	0,014531803	0,063165005	9,106062063	0,5902099	0,417341422
GGO_Jangu	35,460224	5,1428432	2,6046996	0,21295892	0,073454121	0,041408791	6,895062249	0,581405286	0,41111562
GGO_Gaidi	33,658867	4,2064085	0,112613685	0,5191625	0,003345736	0,123421798	8,001806529	0,987960151	0,698593322
GGO_Fritz	30,989285	4,383412	5,38584	0,085255995	0,173796846	0,019449688	7,069671982	1,236356762	0,874236251
PPY_Thai	5,3742967	4,9923315	0,30299538	0,043671217	0,05637861	0,00874766	1,076510384	0,061418378	0,043429351
PPY_Napoleon	4,4169855	4,7949696	0,99302524	0,17000455	0,224819674	0,035454771	0,921170699	0,209656765	0,14824972
MMU_13577	4,5726614	4,617772	0,365866	0,002298183	0,08001161	0,000497682	0,99023109	0,079231516	0,056025142
MMU_13696	4,55268	4,2621994	0,34365967	0,32892582	0,075485136	0,07717279	1,068152748	0,11530928	0,081535974
MNE	4,197667	4,3815165	0,58619034	0,11745585	0,139646699	0,026807123	0,958039756	0,136229825	0,096329033
PHA	5,105591	4,665986	0,5817833	0,31188264	0,113950236	0,066841744	1,094214813	0,144554329	0,102215346

T test(p values)

**HSA vs PTR+GGO**	0,000005677
**HSA vs PPY**	0,000000001
**PTR+GGO vs PPY**	0,000000003
